# Deep-dose: a voxel dose estimation method using deep convolutional neural network for personalized internal dosimetry

**DOI:** 10.1038/s41598-019-46620-y

**Published:** 2019-07-16

**Authors:** Min Sun Lee, Donghwi Hwang, Joong Hyun Kim, Jae Sung Lee

**Affiliations:** 1 Department of Nuclear Medicine, College of Medicine, Seoul National University, Seoul, 03080 Korea; 2 Department of Biomedical Sciences, College of Medicine, Seoul National University, Seoul, 03080 Korea; 3 Center for Ionizing Radiation, Korea Research Institute of Standards and Sciences, Daejeon, 34113 Korea

**Keywords:** Positron-emission tomography, Biomedical engineering

## Abstract

Personalized dosimetry with high accuracy is crucial owing to the growing interests in personalized medicine. The direct Monte Carlo simulation is considered as a state-of-art voxel-based dosimetry technique; however, it incurs an excessive computational cost and time. To overcome the limitations of the direct Monte Carlo approach, we propose using a deep convolutional neural network (CNN) for the voxel dose prediction. PET and CT image patches were used as inputs for the CNN with the given ground truth from direct Monte Carlo. The predicted voxel dose rate maps from the CNN were compared with the ground truth and dose rate maps generated voxel S-value (VSV) kernel convolution method, which is one of the common voxel-based dosimetry techniques. The CNN-based dose rate map agreed well with the ground truth with voxel dose rate errors of 2.54% ± 2.09%. The VSV kernel approach showed a voxel error of 9.97% ± 1.79%. In the whole-body dosimetry study, the average organ absorbed dose errors were 1.07%, 9.43%, and 34.22% for the CNN, VSV, and OLINDA/EXM dosimetry software, respectively. The proposed CNN-based dosimetry method showed improvements compared to the conventional dosimetry approaches and showed results comparable with that of the direct Monte Carlo simulation with significantly lower calculation time.

## Introduction

Internal dosimetry is becoming increasingly important owing to the growing interest in targeted radionuclide therapies, radiotheranostics, and personalized medicine^1–3^. Conventionally, internal dosimetry is conducted using the schema provided by the Medical Internal Radiation Dose (MIRD) committee of the Society of Nuclear Medicine^4–6^. Thus far, organ-based dosimetry is considered as a practical approach to internal dosimetry in nuclear medicine. The organ-based dosimetry calculates organ doses by applying organ-level S-values, which represent the absorbed doses to a target organ per unit activity in a source organ, on the generalized human mathematical phantom. However, organ-based MIRD schema assumes uniform activity distribution in each organ, which is not true. Furthermore, patient-specific body anatomy and tissue composition are not considered.

Therefore, voxel-based dosimetry techniques that consider heterogeneous activity distributions have been suggested, including the dose point kernel^7–9^ and voxel S-value (VSV) approaches^10^. The dose point kernel represents a radial absorbed dose in a homogeneous water medium when an isotropic point source is located at the center^11–15^. The VSV is the voxel-level MIRD schema, in which sources and targets are defined in the voxel-level, and the voxel S-values are calculated in a 3D voxel matrix composed of the water medium. However, the application of the DPK or VSV methods is limited to lesions in homogeneous tissue media (i.e. hepatic ^90^Y-microsphere therapy) because the medium heterogeneity is not considered in these relatively simple analytical approaches.

For more accurate personalized dosimetry, voxel-based dosimetry based on direct Monte Carlo simulation that can consider both heterogeneous activities and medium distributions has been suggested. The Monte Carlo simulation generates and tracks particles at the voxel-level and calculates deposited energy to estimate the voxel-level absorbed doses^16,17^. Nevertheless, this approach requires extensive computational time and resources; hence, it is rarely used in a clinical routine basis. Therefore, it is necessary to develop a fast voxel-based dosimetry technique that takes accounts of heterogeneous activities and medium distribution.

Recently, deep neural networks, which is well-known as deep learning, has gained huge attention in various fields^18,19^. In particular, deep learning approaches outperforms conventional image processing approaches in many different tasks including image classifications, segmentations, and generations^20–27^. Furthermore, there have been some recent attempts to use deep learning techniques for radiation dose estimation^28–30^. However, these deep learning applications are only limited to external radiation therapy.

**Figure 1:**
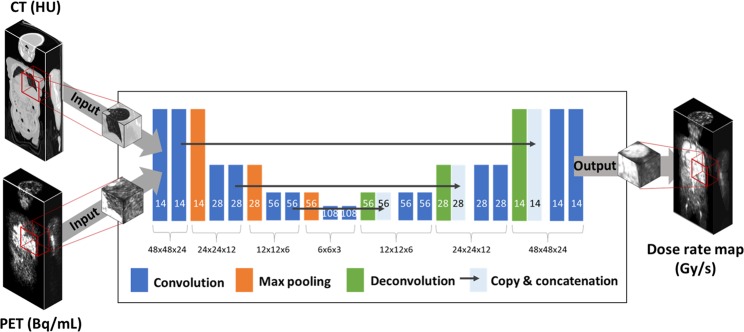
U-net architecture consisting of contracting and expanding path. Each box represents a feature map with corresponding matrix dimension. The number of feature maps is denoted on the bottom of the box.

In this study, we suggest a new internal radiation dose calculation method, called Deep-dose, which applies a convolutional neural network (CNN) to estimate the voxel dose values from the individual nuclear medicine images. The absolute 3D radioactivity distribution given by quantitative positron emission tomography (PET) or single photon emission tomography (SPECT) and media property derived from transmission scans, such as X-ray computed tomography (CT), are fed to the CNN as an input, and the CNN is then trained to generate the dose rate map as an output, with the Monte Carlo simulation based dose rate map as the reference (ground truth). We adopted 3D patch-based network training rather than image-to-image mapping as shown in Fig. 1, considering the range of dose delivery from a source voxel to surrounding tissues. To show the feasibility of the Deep-dose method, we applied it to the PET/CT data set of ^68^Ga-NOTA-RGD, which is a promising diagnostic PET agent for angiogenesis assessment. We evaluated the performance of the Deep-dose by comparing its results to those of direct Monte Carlo based voxel dose estimation.

## Methods

### Patient data set

In this study, patient datasets (10 subjects; four females and six males; 53.7 ± 13.5 years; 61.5 ± 7.4 kg) acquired in our previous study were used retrospectively^31^. All procedures in this study were approved by the Institutional Review Board of Seoul National University Hospital, Seoul, Korea and performed in accordance with relevant guidelines and regulations. The retrospective use of the scan data and waiver of consent were approved by the Institutional Review Board of our institute. Dynamic PET/CT images at eight time points were acquired after an intravenous injection of ^68^Ga-NOTA-RGD. The durations of emission scans varied from 30 to 300 seconds per bed, and the scans were started at 1, 4, 7, 10, 15, 30, 46, and 62 min post-injection. The detailed PET/CT imaging protocol is described by Kim *et al*.^31^. The reconstructed PET images had dimensions of 256 × 256 × 165 with a voxel size of 2.67 × 2.67 × 5 mm3. The regions-of-interests were manually drawn on the CT images for eight organs: gallbladder wall, heart, kidneys, liver, lungs, pancreas, spleen, and stomach wall. All region-of-interests were drawn by a single individual (nuclear medicine physicist) and confirmed with other nuclear medicine physicists.

### Dose estimation using direct Monte Carlo simulation

Dose estimation using the direct Monte Carlo simulation was considered as the ground truth data. The Geant4 Application for Emission Tomography (GATE) v.7.0 simulation toolkit^32,33^ was used to generate the ground truth dose maps. The patient PET and CT images were imported to simulate the voxelized source distribution and voxelized phantom. The CT images were matched with the PET images to have the same matrix and pixel sizes. CT Hounsfield units were converted into densities based on the published database^34^. The standard electromagnetic physics package of Geant4 v.9.6.3 was used for simulating the ^68^Ga ion particles and photon interactions at each voxel with specific material composition. The dose calculation mechanism called “Dose Actor” was used for voxel dose calculation^33^. An in-house computing cluster with a 60-cores of Intel XEON 2.4 GHz CPU and 80 GB DDR3 RAM was used for the simulation. Owing to the long simulation time and extensive computational cost for the Monte Carlo simulation, the simulation was conducted only for 5% of the real scan duration of each PET frame. Although the simulation was performed only for 5% of actual events, the statistical uncertainties of the dose estimation at the voxel-level was less than 0.1% for all voxels. The generated 3D dose maps (Gy = J/kg) from the GATE simulation were divided using the corresponding simulation times to generate the 3D dose rate maps (Gy/s) and used as reference data for the CNN.

### Dose estimation using voxel S-value kernel convolution

For the comparison, we used the VSV kernel convolution method to calculate voxel doses. The VSV (Gy/MBq·s) was generated using the previously described GATE simulation set up while placing the ^68^Ga point source at the center of the water medium. The VSV kernel had the same voxel size as the PET image. The time-integrated activity ($\tilde{A}$) (MBq·s) was convoluted with the VSV kernel to generate the voxel dose (${D}_{voxel}$) (Gy) as shown by the equation below.

$${D}_{voxe{l}_{j}}=\tilde{A}\otimes VSV=\sum _{i=0}^{n}{\tilde{A}}_{voxe{l}_{i}}\times VSV(voxe{l}_{j}\leftarrow voxe{l}_{i})$$

### Voxel dose estimation using deep convolutional neural network

#### Network architecture

The deep neural network used for the voxel dose estimation is based on U-net structure^25^ that has a large receptive field and shown remarkable performance in various image processing tasks including image segmentation, restoration, and denoising^23,35^. Because the dose of a voxel is determined by the surrounding source and the medium distributions, we performed 3D patch-based training of U-net (Fig. 1). We designed the network to predict the dose rate map using the PET and CT image patches as inputs after training the network using the Monte Carlo simulation-based ground truth dose rate map as a reference. Each image patch had a matrix size of 48 × 48 × 24. We determined the matrix size to be large enough to cover the dose range of ^68^Ga to avoid the underestimation of the absorbed dose^36^.

Figure 1 shows the U-net network architecture^24^, consisting of a contracting and expanding path. The contracting path consists of the 3 × 3 × 3 convolution layer each followed by batch normalization, rectified linear units as an activation function, and a 2 × 2 × 2 max pooling layer. The expansion path consisted of 3 × 3 × 3 convolution and deconvolution, each followed by rectified linear units and recovered feature maps into high-resolution images by copying and concatenating the feature maps from the contracting path. The number of feature maps in the first layer was empirically set to 14, then doubled in the contracting path, and finally reduced by half in the expanding path.

#### Network training and testing

The network was trained and tested by the fashion of five-fold cross-validation. In each cross-validation, eight out of the ten patient datasets were used for network training and the remaining two datasets were used to test the performance of the proposed CNN approach. Therefore, all patient datasets were used for network training four times and exactly once for testing.

To increase the number of training data, one static PET/CT image set with a matrix size of 256 × 256 × 165 was divided into 3D image patches with a matrix size of 48 × 48×24 (patch-based learning). In order to keep the dose contribution from the outside of the given image patch, image patches were generated with overlaps with 7 pixel offsets in the transverse direction and 5 pixel offsets in the axial direction. Total 5,000 image patches were generated from a single static image, while image patches which contained less than a one eights of the imaged body part were excluded. Therefore, the total number of training dataset were 8 (number of patients) × 8 (number of time points) × 5,000 (number of image patches) = 320,000 each for PET and CT inputs.

The cost function was L1-norm between the ground truth dose rate map and the dose rate map predicted by the CNN. The cost function was minimized using the adaptive moment (ADAM) estimation method. The batch size for batch normalization was 150 and the number of epochs was seven. The network was implemented using TensorFlow under the computational environment with Intel Core i5-2500 CPU and GTX 1080 Ti GPU.

The dose rate maps generated using the trained CNN were compared to the ground truth dose rate map obtained using direct Monte Carlo simulation. The dose rate maps estimated by the VSV kernel convolution method were also compared. Errors at the voxel-level were analyzed in terms of relative and absolute differences and represented in the 3D difference map. Mean voxel-level dose difference at eight different organs were reported by calculating the mean value of absolute dose % differences in each organ. Also, we conducted paired *t*-test between voxel-level dose rates of the ground truth and VSV- and CNN-based dose estimation, respectively. Bonferroni correction was applied to adjust levels of significance for multiple t-tests. The test rejected the null hypothesis at the 5% significance level.

Finally, whole-body dosimetry was conducted using the proposed CNN-based approach, VSV kernel convolution, and organ-based dosimetry approach using OLINDA/EXM software^6^. The outcome of these three different approaches were compared with the ground truth. The dose rate maps at each time point were integrated by the trapezoidal sum of dose rate until the last time point (62 min) and extrapolation of the tail to the infinity to acquire absorbed doses. Absorbed doses for each organ were corrected using the organ masses of the Oak Ridge National Laboratory stylized phantoms for the fair comparison with the organ-based dosimetry results published by Kim *et al*.^31^. Paired *t*-test was conducted between absorbed organ doses of the ground truth and VSV-, CNN-, and MIRD-based absorbed dose, respectively.

## Results

### Whole-body dose rate maps

**Figure 2:**
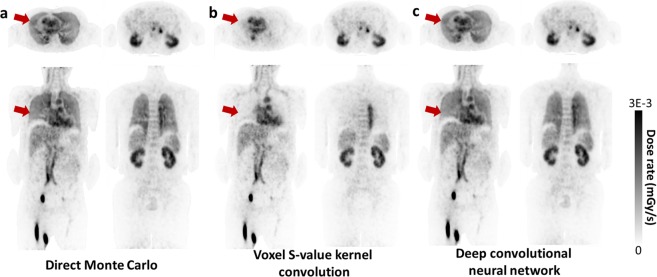
Dose rate maps estimated by (**a**) direct Monte Carlo, (**b**) VSV kernel convolution, and (**c**) deep convolutional neural network.

The whole-body dose rate maps generated by direct Monte Carlo simulation, which is considered as the ground truth, were compared with conventional VSV kernel convolution and the proposed approach. As shown in Fig. 2, the proposed CNN approach yielded dose rate maps that agreed well with the ground truth. Moreover, the CNN approach showed remarkably better results in lungs compared to the VSV kernel method (red arrows in Fig. 2).

**Figure 3:**
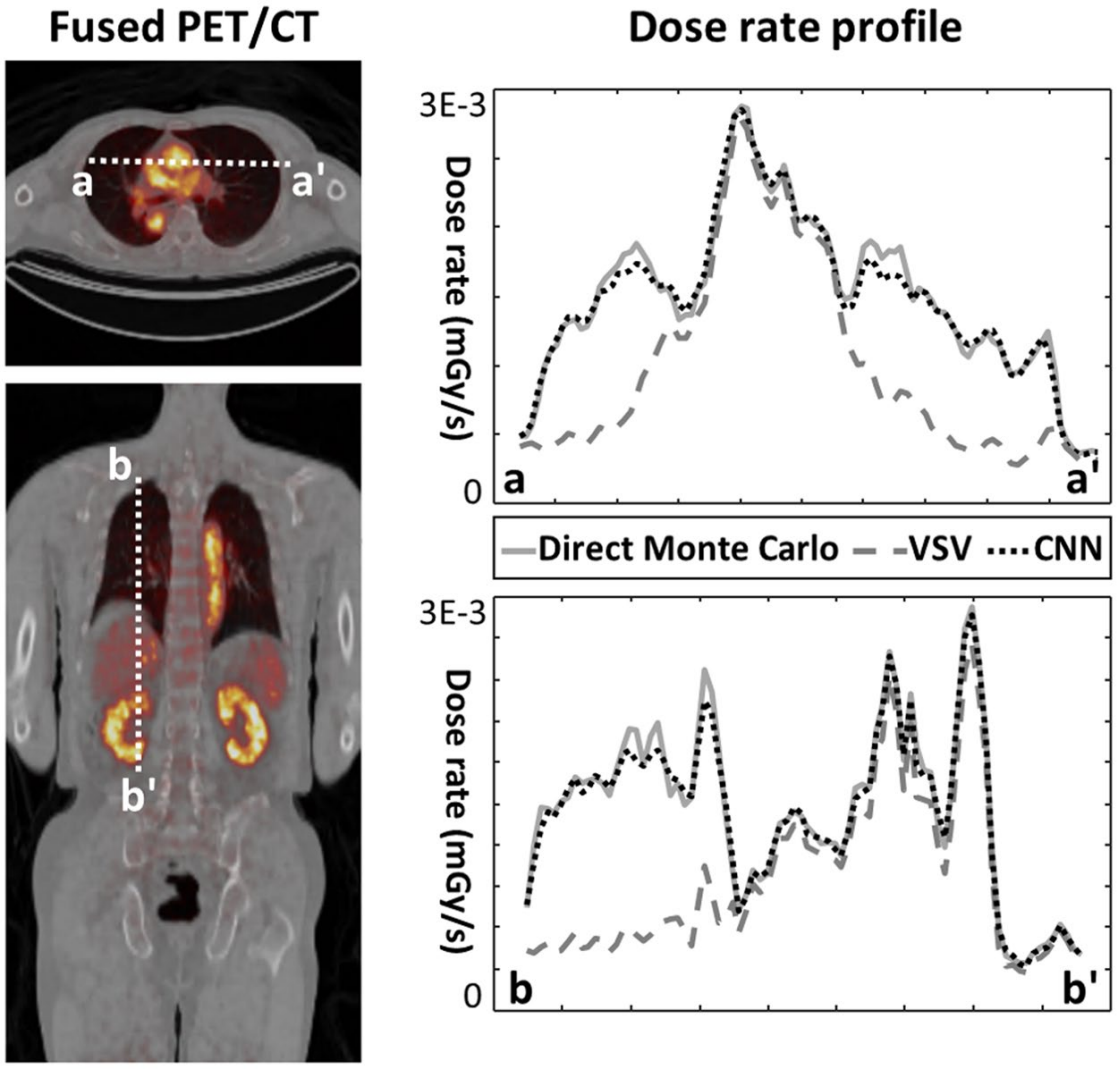
Two dose rate profiles drawn on the axial and coronal slices.

Figure 3 shows the dose rate profile of each method along the coronal and axial slices. The VSV approach showed a similar profile with the direct Monte Carlo simulations on the soft tissue regions (heart, liver, and kidneys) while significant underestimation of dose for the lungs was observed owing to the medium density mismatch in the VSV kernel. However, the results of the proposed approach matched very well with the direct Monte Carlo approach for all regions regardless of the medium density.

### Voxel-level dose rate error

**Figure 4:**
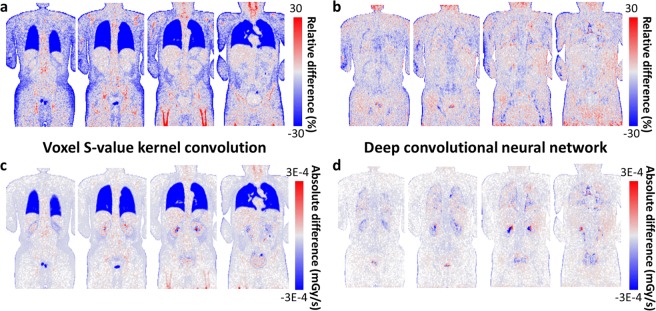
Relative voxel errors of (**a**) VSV kernel convolution and (**b**) CNN approach presented in 2D maps for representative coronal slices. Absolute voxel errors of (**c**) VSV kernel convolution and (d) CNN approach for the same coronal slices.

To analyze the dosimetric accuracy quantitatively, we calculated errors at the voxel-level and generated 3D maps of relative and absolute differences of the dose rate. Figure 4 shows four coronal slices of a representative case. The VSV kernel convolution approach yielded low errors in the soft tissue regions while those of the lungs, bone, and air cavity regions had high errors due to the VSV density mismatch as shown in Fig. 4(a,c). The CNN approach showed low and uniform errors in both the relative and absolute difference map as shown in Fig. 4(b,d).

**Figure 5:**
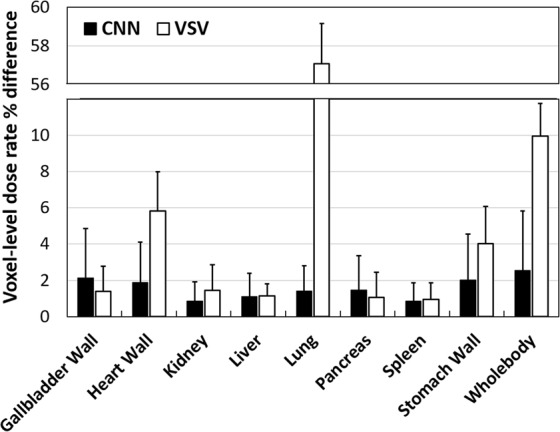
Mean voxel dose rate errors (%) reported for the CNN and VSV approach for eight different organs and the whole-body. Mean percentage differences of each method are denoted via rectangular bars and standard deviations are denoted using error bars.

Figure 5 and Table 1 summarizes the voxel-level dose rate errors for the VSV and CNN approach for eight organs and the whole-body. The VSV kernel approach showed low voxel-level errors except for the lung with an average voxel error was 9.97% ± 1.79% at the whole-body level. However, based on the *t*-test results, we observed half of the organs showed significant difference with the direct Monte Carlo results. On the other hand, the CNN approach showed reliable dose rate prediction. Eight organs showed very low voxel-level errors and most of the organs except for lung showed non-significant difference with the direct Monte Carlo approach. With the CNN approach, voxel dose rate percentage errors of 2.54% ± 2.09% was achieved at the whole-body level.

**Table 1:** Voxel-level dose rate errors (mean ± std) of static patient images (*n* = 80) and their differences to the ground truth were statistically analyzed with paired *t*-test.

$${\bf{M}}{\bf{e}}{\bf{a}}{\bf{n}}\,{\bf{v}}{\bf{o}}{\bf{x}}{\bf{e}}{\bf{l}}\,{\textstyle \text{-}}\,{\bf{l}}{\bf{e}}{\bf{v}}{\bf{e}}{\bf{l}}\,{\bf{d}}{\bf{o}}{\bf{s}}{\bf{e}}\,{\rm{ \% }}\,{\bf{d}}{\bf{i}}{\bf{f}}{\bf{f}}{\bf{e}}{\bf{r}}{\bf{e}}{\bf{n}}{\bf{c}}{\bf{e}}=\,{{\boldsymbol{\sum }}}_{1}^{{\boldsymbol{n}}}|\frac{{\boldsymbol{M}}{\boldsymbol{e}}{\boldsymbol{t}}{\boldsymbol{h}}{\boldsymbol{o}}{{\boldsymbol{d}}}_{{\boldsymbol{n}}}^{\ast }-{\boldsymbol{D}}{\boldsymbol{M}}{{\boldsymbol{C}}}_{{\boldsymbol{n}}}^{{\rm{\S }}}}{{\boldsymbol{D}}{\boldsymbol{M}}{{\boldsymbol{C}}}_{{\boldsymbol{n}}}}|/{\boldsymbol{n}}\times {\bf{100}}$$		
Method	VSV	Deep-dose
	Mean ± Std (%)	Mean ± Std (%)
Gallbladder wall	1.38 ± 1.37	2.13 ± 1.70
	*NS*	*NS*
Heart wall	5.83 ± 2.16	1.88 ± 1.27
	*p* < 0.005	*NS*
Kidney	1.44 ± 1.14	0.86 ± 0.67
	*NS*	*NS*
Liver	1.13 ± 0.68	1.10 ± 0.67
	*NS*	*NS*
Lung	57.09 ± 2.07	1.41 ± 1.29
	*p* < 0.001	*p* < 0.05
Pancreas	1.05 ± 0.98	1.47 ± 1.21
	*NS*	*NS*
Spleen	0.94 ± 0.68	0.85 ± 0.61
	*p* < 0.01	*NS*
Stomach wall	4.01 ± 2.02	2.01 ± 1.61
	*p* < 0.05	*NS*
Whole-body (avg)	9.97 ± 1.79	2.54 ± 2.09

The degree of significance is reported in the below of the corresponding differences (*NS* = nonsignificant).

*Method: VSV or Deep-dose (CNN). ^§^DMC: direct Monte Carlo simulation.

### Computation time

Training the CNN took approximately 18 hr for each epoch, and a total 126 hr for training 7 epochs. Using the trained CNN network, single dose rate maps can be generated in just 3.6 min with PET and CT input images. On the contrary, the direct Monte Carlo simulation took 235.2 hr for generating single dose rate map using a CPU with four cores and 16 GB RAM. Using the proposed CNN-based voxel dose rate estimation, we could reduce the computation time for 3D dose rate map generation. Table 2 summarizes the computation time for the single 3D dose rate map generation. However, we would like to note that the direct Monte Carlo simulation and CNN network was implemented in the different computation environment, and there is some room for speeding up the direct Monte Carlo simulation by implementing GPU-based Monte Carlo. However, the CNN-based voxel-dose calculation is still significantly faster than the direct Monte Carlo.

**Table 2:** Computation time for generating a single 3D dose rate map.

Method	Time consumption (hr)	Relative time consumption
Direct Monte Carlo (5% of actual events)	235.20	1
Direct Monte Carlo (complete simulation)	4704.03	20
VSV	0.17	0.00074
Deep-dose (CNN)	0.06	0.00026

### Whole-body dosimetry in patients

**Figure 6:**
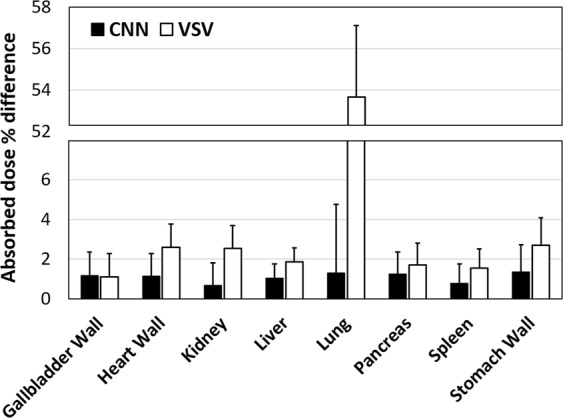
Results of whole-body patient dosimetry studies for 10 patients. Mean absorbed dose percentage differences (%) were reported for the CNN and VSV approaches for eight different organs. Mean percentage differences of each method are denoted by rectangular bars and standard deviations are denoted using error bars.

Finally, the dosimetry study was conducted using the dynamic PET/CT images of 10 patients. Figure 6 and Table 3 shows the result of the whole-body dosimetry study. Our proposed method yielded better results than the conventional VSV kernel convolution and organ-based dosimetry. For the proposed CNN approach, errors at the organ level were observed under 1.5%, while VSV approach showed slightly larger organ level errors except for the lung, which showed significantly larger errors. Organ-based MIRD approach showed very large errors compared to voxel-based ground truth data. The average organ dose percentage difference was 1.07%, 9.43%, and 34.22% for the CNN, VSV, and organ-based dosimetry results, respectively. Based on the paired *t*-test results between the direct Monte Carlo and three different dose calculation approaches, respectively, the CNN approach showed non-significant differences in absorbed dose estimation while VSV and organ-based dosimetry approach showed significant difference in most of the organs.

**Table 3:** Whole-body patient dosimetry results reported in organ absorbed dose difference (mean ± std) for ten patients (*n* = 10) and their differences to the ground truth were statistically analyzed with paired *t*-test.

$${\bf{M}}{\bf{e}}{\bf{a}}{\bf{n}}\,{\bf{o}}{\bf{r}}{\bf{g}}{\bf{a}}{\bf{n}}\,{\bf{a}}{\bf{b}}{\bf{s}}{\bf{o}}{\bf{r}}{\bf{b}}{\bf{e}}{\bf{d}}\,{\bf{d}}{\bf{o}}{\bf{s}}{\bf{e}}\, \% \,{\bf{d}}{\bf{i}}{\bf{f}}{\bf{f}}{\bf{e}}{\bf{r}}{\bf{e}}{\bf{n}}{\bf{c}}{\bf{e}}={{\boldsymbol{\sum }}}_{1}^{n}|\frac{{\boldsymbol{Metho}}{{\boldsymbol{d}}}_{{\boldsymbol{n}}}^{\ast }-{\boldsymbol{DM}}{{\boldsymbol{C}}}_{{\boldsymbol{n}}}^{\S }}{{\boldsymbol{DM}}{{\boldsymbol{C}}}_{{\boldsymbol{n}}}}|/{\boldsymbol{n}}\times {\bf{100}}$$			
Method	VSV	Deep-dose	Organ-based dosimetry
Gallbladder wall	1.12 ± 1.17	1.18 ± 1.20	22.27 ± 22.28
	*NS*	*NS*	*p* < 0.001
Heart wall	2.60 ± 1.15	1.14 ± 1.09	76.39 ± 27.05
	*p* < 0.05	*NS*	*p* < 0.001
Kidney	2.55 ± 1.14	0.67 ± 0.57	3.86 ± 3.29
	*p* < 0.05	*NS*	*p* < 0.001
Liver	1.86 ± 0.71	1.04 ± 0.99	19.01 ± 31.90
	*p* < 0.01	*NS*	*p* < 0.001
Lung	53.66 ± 3.45	1.30 ± 1.15	23.43 ± 33.86
	*p* < 0.001	*NS*	*p* < 0.001
Pancreas	1.71 ± 1.09	1.26 ± 1.74	1365.72 ± 1186.49
	*NS*	*NS*	*p* < 0.001
Spleen	1.55 ± 0.97	0.79 ± 0.77	46.62 ± 104.20
	*NS*	*NS*	*p* < 0.001
Stomach wall	2.70 ± 1.38	1.35 ± 1.19	42.94 ± 71.27
	*p* < 0.05	*NS*	*p* < 0.001
Avg.	8.47 ± 1.38	1.09 ± 1.09	200.66 ± 185.04
Avg. (excluding Pancreas)	9.43 ± 0.85	1.07 ± 0.22	34.22 ± 31.63

^*^Method: VSV or Deep-dose (CNN). ^§^DMC: direct Monte Carlo simulation.

The degree of significance is reported in the below of the corresponding differences (*NS* = nonsignificant).

## Discussion

Whole-body voxel-based dosimetry techniques that use real patient anatomy and activity distributions have several advantages over conventional organ-based dosimetry. By accounting real patient information, more accurate dose information can be calculated for an individual, which is very important in personalized medicine for the risk-benefit assessments. Moreover, the 3D voxel dose distribution given by voxel-based dosimetry enables the assessment of dose volume histogram in targets. However, the current voxel-based dosimetry approaches have a limitation of dose calculation time, so rarely used in clinical practice. Therefore, fast whole-body voxel-based dosimetry technique will play a role as a powerful dosimetry toolkit and truly introduce personalized medicine in clinical routines.

In this study we used deep learning to generate the dose rate map from PET and CT images. We used deep learning as a mean of exploring the relationship between heterogeneous activity/media distributions and the deposited energies at each corresponding voxel. We used the 3D image patch-based learning for the data augmentation and the size of image patch was determined as the size of the dose kernel of a voxel S-value to avoid underestimation of energy deposition by including a sufficient energy deposition range.

Based on our result of CNN-based voxel dose estimation, we conclude that the proposed method is a promising method for voxel-based dosimetry. The difference between CNN-based dose rate map and the ground truth image was relatively small. When compared to the conventional VSV kernel convolution, the CNN outperformed on voxel dose rate estimation especially in the lungs, bones, air cavity, and in the organ boundaries as shown in Fig. 3 and Table 1. For most of the body parts, we did not observe any dose estimation bias in the image patch-based CNN approach except for the bladder, which contains ultra-high activities that leads to image artifacts in the PET image itself, we observed dose estimation bias. However, it is hard to say that this bias is caused by the CNN, because it was observed in the VSV approach as well. Finally, whole-body dosimetry study was conducted and the results of the dosimetry study (Table 3) showed that the CNN-based approach exhibited only small errors in organ dose calculation while the VSV approach exhibited large underestimation in the lung regions. The organ-based dosimetry approach showed even larger differences throughout the organs because this method is not appropriate for personalized dosimetry. Moreover, in terms of computation time, the CNN approach showed significant improvement in the time required for dose rate map generation compared to the direct Monte Carlo based voxel dose estimation as shown in Table 2.

The main limitation of this study is the small patient population (10 patients). Because our data augmentation strategy relied on the patch-based training and the use of eight time points, the data from multiple time points would be correlated thus limiting the additional information fed to the CNN. Although we tried to reduce the data correlation due to multiple data samples from the same individual by random data shuffling, validation of our proposed method with the large independent dataset is ultimately necessary. We warrant further validation studies with much larger population data and various radiotracers in the future.

Moreover, to implement our proposed method to PET or SPECT images that are acquired with other radioisotopes, additional network training is required because different isotopes have different emissions schemes with different energy values. In the proposed CNN architecture, the labeled image is required because supervised learning is used and hence the direct Monte Carlo based dose map generation is also required. Given that this ground truth dose map generation takes significantly long time, further study is required to figure out alternative methods, such as to use the labeled dose map that is generated by multiple VSV kernels^37^, which is much faster than Monte Carlo simulation. With the trained network, the CNN-based voxel dose map generation would be a powerful tool for the personalized dosimetry technique that realizes personalized medicine just in a few minutes. It is a bit early to say that the CNN-based approach can substitute the sophisticated nature of Monte Carlo engine; however, our result reports that CNN can be considered as a candidate for a fast voxel-based dosimetry technique.
